# Quantity and Quality of Evidence Are Sufficient: Prevalent Features of Ultraprocessed Diets Are Deleterious for Health

**DOI:** 10.1016/j.advnut.2023.100157

**Published:** 2023-12-18

**Authors:** Kenny Mendoza, Deirdre K Tobias

**Affiliations:** 1 Department of Nutrition, Harvard T.H. Chan School of Public Health, Boston, MA, United States; 2 Division of Preventive Medicine, Department of Medicine, Brigham and Women's Hospital and Harvard Medical School, Boston, MA, United States

The United States is a leading consumer of ultraprocessed foods (UPFs) globally, with a staggering 55% of adults’ energy intake coming from these products [1]. In addition to enhanced processing techniques, Nova-defined UPFs also contain cosmetic additives to enhance palatability, profitability, and shelf-life, at no nutritional value [2]. Recent meta-analyses documented significant adverse associations of UPFs with diabetes [3] and cardiovascular disease [4]. Building on this growing body of literature, Vitale et al. [5] conducted an comprehensive systematic review, highlighting the following 3 salient conclusions: *1*) heterogeneity in risk estimates is not as profound as some UPF industry and other stakeholders argue; *2*) UPF classification should evolve to reflect both food processing and nutritional value; and *3*) although the quality of UPF research can (and should) be improved on, limitations of the current evidence base are not insurmountable, nor do they serve as sole justification to delay public health interrogation around UPFs.

On the first point, Vitale et al. [5] concluded that the highest UPF intake category (compared with the lowest) was associated with a higher risk of obesity and hypertension (32%), hypertriglyceridemia (47%), low HDL-cholesterol (43%), and diabetes (37%). They raised concerns regarding heterogeneity in these summary estimates induced by the type of studies’ dietary assessment instruments. They stratified summary statistics by food-frequency questionnaires compared with 24-h dietary recall methods; for instance, the relative risk for diabetes was 1.53 [95% confidence interval (CI): 1.31, 1.79] among studies with food-frequency questionnaires and 1.25 (95% CI: 1.11, 1.42) for 24-h recalls. There are several other plausible methodologic drivers of between-study heterogeneity for these outcomes, including assessment of diet at baseline only compared with leveraging repeated measures over time, follow-up duration, baseline health status and age, background diet of the population, and outcome ascertainment methods. Although Vitale et al. [5] did not formally test for statistical effect modification between diet assessment instrument types with meta-regression analyses, visual inspection of forest plots and 95% CIs surrounding the point estimates do not indicate significant differences. In sum, between-study heterogeneity was not severe, and importantly, all studies were in the similar direction of association in Vitale et al.’s [5] meta-evidence. Although systematic reviews are often strengthened by including all available studies, this approach risks incorporating lower-quality research designs prone to bias that undermine overall certainty and evidence grading.

Pertaining to the second point, Nova categorizes food products by processing-related factors and without attention to nutritional composition qualities or healthfulness [2]. As such, UPFs range from mass-produced whole-grain vitamin-fortified breads to nutritionally devoid soft drinks. On this matter, Vitale et al. [5] emphasized a critical evidence gap: many original studies do not report the foods and beverages contributing to total UPF intake, nor the relationship of UPF subtypes with health outcomes. For example, in 3 large United States cohorts [3], although artificially and sugar-sweetened beverages, processed meats, and ready-to-eat dishes were associated with higher type 2 diabetes risk, ultraprocessed whole-grain bread, breakfast cereal, and yogurt displayed inverse associations [3]. We observed similar divergent trends for UPFs with cardiovascular disease in the same cohorts [6]. These analyses underscore the feasibility of epidemiologic data to contribute to disentangling the variability in health effects across UPFs. Emerging Nova-specific dietary intake instruments [7] may also contribute to this important direction of UPF research. Furthermore, research of UPF subtypes in culturally diverse cohort studies are needed, particularly where foods’ nutritional composition and quality may differ from the existing evidence base, because these data may inform tailored UPF interventions and policies.

Third, an accumulation of well-designed studies is essential to guide nutritional recommendations. Vitale et al. [5] graded the UPF meta-evidence base utilizing NutriGrade [8] and categorized the synthesis across health outcomes as low to moderate quality. However, important considerations when applying NutriGrade warrant discussion, because their evidence assessments may indicate field-wide challenges that are not unique to UPFs. As the authors note, many of the original studies did not report risk estimates for UPF exposure as a continuous variable or did so with various incompatible units of intake (e.g., servings/day, relative caloric, or weight contributions/day), precluding robust dose-response meta-analyses; as such, points were subtracted during the NutriGrade scoring [8]. Given the lack of standardized categories, thresholds, or intake units for UPFs, investigators should provide various expressions of their exposure-outcome associations to facilitate future meta-analyses. Furthermore, NutriGrade allots points for larger effect estimates above specific thresholds (>1.20 to 2.0 for at least 1 point earned) [8], and penalizes modest magnitudes of association for their potential implausibility, regardless of triangulation of high quality evidence from other research domains such as experimental studies and clinical trials [e.g., comparing extreme UPF intakes, the combined risk of coronary artery disease is 1.18 (95% CI: 1.08, 1.30)] [6]. Therefore, standardized quality assessment tools must ultimately rely on subject matter knowledge to judge the certainty and validity of evidence implicating UPFs.

One controlled feeding trial demonstrates that nonnutritive properties of UPFs contribute to excess energy intake and weight gain [9], corroborated by accumulating observational evidence for an adverse role of UPFs with the development of obesity and poor cardiometabolic health [5]. Although public health will continue to learn from the evolving research landscape as it disentangles the relevant features and pathophysiologic mechanisms of exposure to UPFs, the perceived inconsistency of the current evidence is overstated and largely explained by research methodology rather than uncertainty of true biological harm [10]. Nutrient-centric and Nova-based studies collectively provide sufficient evidence to critically evaluate strategies for meaningful improvements in the UPF supply to mitigate their detrimental health effects. This includes an emphasis on reducing exposures with no nutritional value and consistently associated with harm, such as sugary beverages and ultraprocessed meats, and further pinpointing the precise targets of reformulation. Potential downstream strategies include taxation, restricting prominent positioning and displays in stores, and/or regulating marketing to protect vulnerable populations. The diversity of nutritional quality within UPFs suggests that modifications to this classification can be made for research purposes to better characterize the vast landscape of food processing techniques and ingredients with-respect-to their effects on obesity and health. Further research will undoubtedly uncover the specific high-risk features of UPFs and effective and long-term reformulation strategies to improve population health are in sight.

## Conflict of interest

The authors report no funding or other financial conflicts of interest related to this article.
